# Importance of Hydrophilic Groups on Modulating the Structural, Mechanical, and Interfacial Properties of Bilayers: A Comparative Molecular Dynamics Study of Phosphatidylcholine and Ion Pair Amphiphile Membranes

**DOI:** 10.3390/ijms19061552

**Published:** 2018-05-23

**Authors:** Ching-an Tian, Chi-cheng Chiu

**Affiliations:** 1 Deparment of Chemical Engineering, National Cheng Kung University, Tainan 70101, Taiwan; happy122025@gmail.com; 2 Hierarchical Green-Energy Materials (Hi-GEM) Research Center, National Cheng Kung University, Tainan 70101, Taiwan

**Keywords:** ion pair amphiphile, phosphatidylcholine, bilayer, molecular dynamics

## Abstract

An ion pair amphiphile (IPA), a molecular complex composed of two oppositely charged amphiphiles, is a phospholipid mimic which differs from a phospholipid only in the hydrophilic compositions. Here, we utilized molecular dynamics (MD) simulations to compare the bilayer systems composed of phosphatidylcholines (PC) and alkyltrimethylammonium-alkylsulfate IPAs with various alkyl chain lengths. The membrane properties for both liquid-disordered (Ld) and gel (S) phase bilayers were examined via running simulations above and below the main transition temperatures. The electrostatic attraction between the IPA hydrophilic groups leads to a more ordered molecular packing within both S and Ld phase IPA membranes, as revealed by the molecular area, deuterium order parameter, and gauche conformation analyses. Furthermore, IPA bilayers possess a higher area compressibility modulus, molecular tilt modulus, and effective bending rigidity than PC systems. The variation of hydrophilic groups of IPA also leads to fewer hydrogen bonds on the membrane surface and smaller electrostatic potentials for the biomimetic bilayer. The non-covalently linked head groups of IPA further decouple alkyl tilting and surface water retention. The combined results reveal the importance of hydrophilic groups of amphiphiles on modulating the membrane properties, which also provides insights for designs of biomimetic membranes.

## 1. Introduction

Phospholipids are natural occurring amphiphiles that are the major components of cell membranes. Its main functions include maintaining the spatial orientations of organelles, providing barriers to distinguish the cytoplasm from the extracellular surrounding and separating various biochemical reactions inside the cell [1]. A phospholipid, like a common amphiphilic molecule, contains a hydrophobic part that includes two hydrocarbon chains and a hydrophilic head that is usually composed of a negatively charged phosphate group, a functional group attaching to the phosphate, and a diglyceride linking with two alkyl chains [2]. The variations of the functional group result in different phospholipid types. For instance, a phosphatidylcholine (PC), one of the most common phospholipids, has a positively charged choline attached to the phosphate, whereas a phosphatidylethanolamine (PE) contains an ethanolamine group instead. Both PC and PE have an overall neutral charge and hence are zwitterionic. In contrast, phophatidylserine (PS) has as neutral serine group linked with the phosphate and thus has an overall negative charged. Phospholipids have been shown to self-assemble in vitro into micro-structures such as bilayers and vesicles [3,4]. Lipid bilayers, the main structures of the cell membranes, are made of two leaflets of lipid monolayers with the hydrophobic tails buried at the bilayer center. Wrapping a bilayer into a hollow spherical form results in a vesicle. Liposomes, vesicles composed of phospholipids, have attracted much attention due to their potentials in drug deliveries and gene therapies [5,6,7].

Other than phospholipids, vesicles can be formed using two oppositely charged single-chained amphiphiles, as first demonstrated by Kaler et al. with the mixture of hexadecyltrimethyl-ammonium tosylate and sodium dodecylbenzene sulfonate [8]. The resulting vesicles are termed catanionic vesicles. Further removing the counter ions from the equal-molar mixtures of cationic and anionic amphiphiles produces a complex called an ion pair amphiphile (IPA) [9,10,11]. The two amphiphilic components of an IPA are held together via the electrostatic attraction between the two oppositely charged hydrophilic head groups. An IPA composed of cationic and anionic amphiphiles, each with a single alkyl chain, is therefore pseudo-double-tailed and a mimic of a zwitterionic phospholipid such as PC and PE. The vesicles prepared from IPAs usually exhibit low colloidal stability [12,13,14]. The stability can be improved inter-vesicular using additives such as charged amphiphiles or polymers that increase the electrostatic or steric repulsion among IPA vesicles [15,16,17]. Meanwhile, additives such as cholesterol have been shown to improve the intra-vesicular stability of IPA vesicles via strengthening the mechanical properties of the vesicular bilayer [18,19,20,21,22]. The vesicles prepared from biomimetic IPAs are therefore good candidates as phospholipid substitutes and have great potentials in various applications such as cosmetics and drug carriers [23,24].

At a given temperature, a lipid bilayer can exist in either a gel or liquid-disordered phase, and each type of lipid has a characteristic temperature, T${}_{m}$, at which the bilayer transitions from the gel to the liquid-disordered phase [1,25]. The gel (S) phase is commonly referred to as a solid-like phase with most alkyl chains aligned; while in the liquid-disordered (Ld) phase, lipid molecules can move more freely resulting in more disordered hydrophobic chains. The phase behavior of a lipid bilayer is largely determined by the strength of the van der Waals (vdW) interaction among the alkyl chains: longer-tailed lipids have stronger vdW interactions resulting in higher T${}_{m}$ [26,27]. Saturated PC with two alkyl tails longer than 14 carbons are in the gel phase at 298 K, while those with two alkyl chains fewer than 14 carbons are in the fluid state [26,28]. The T${}_{m}$ of saturated PC also gradually increases with the alkyl chain elongation. The phase behavior of bilayers composed of alkyltrimethylammonium-alkylsulfate IPA also shows the similar alkyl chain length to T${}_{m}$ relation [27]. This suggests a close structural correlation between the IPA and the saturated PC bilayer system.

**Figure 1:**
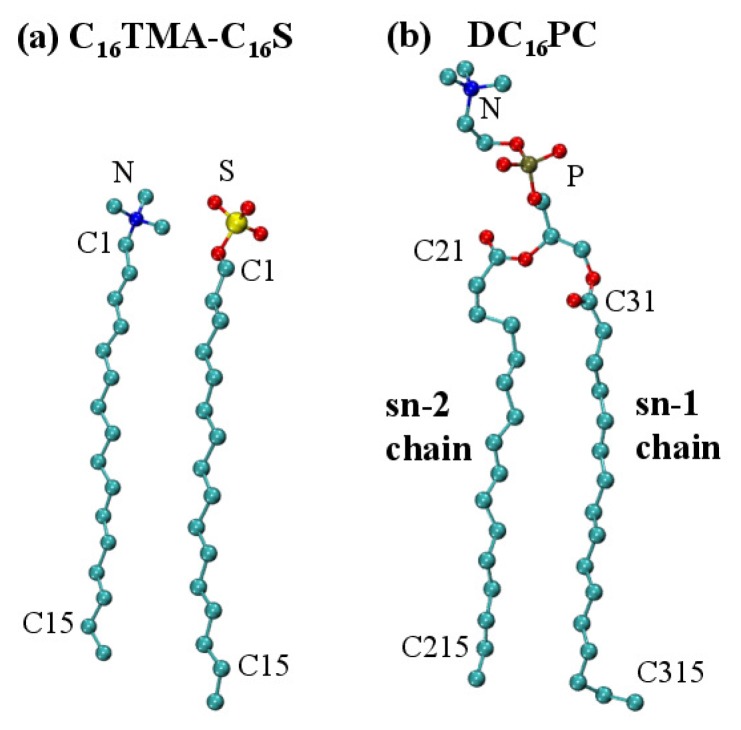
Molecular structures of (**a**) C${}_{16}$TMA-C${}_{16}$S IPA complex, and (**b**) DC${}_{16}$PC. The atoms utilized for data analyses are also labeled. The color codes for atoms are nitrogen in blue, sulfur in yellow, oxygen in red, and carbon in turquoise. Hydrogen atoms are not shown for clarity. Graphics were generated using VMD [32].

Comparing the molecular structures of PC and IPA, the main difference is the composition of the hydrophilic groups. The hydrophilic part of a PC consists of choline, phosphate, and glyceride, whereas that of an IPA complex includes two oppositely charged groups, e.g., trimethylamine and sulfate groups in Figure 1. The hydrophilic group is also used to categorize phospholipid, such as PC, PE, and PS. Various hydrophilic groups can lead to diverse structural and thermodynamics characteristics of the resulting bilayers. For instance, PC and PE only differ in the choline and ethanolamine groups. Yet, a PE bilayer exhibits a smaller area per lipid than the PC system [29,30]. Additionally, the T${}_{m}$ value for a PE bilayer is higher than that for the PC bilayer with the same alkyl chain composition [26]. These variations can be correlated with the ethanolamine group of PE that can form H-bonds with phosphate groups intra-molecularly and with surrounding waters [29,30].

Other than T${}_{m}$ data, however, there have been very limited studies focusing on the comparison of the membrane properties between phospholipid and IPA bilayer systems. Here, we applied molecular dynamics (MD) simulations to study the bilayer systems composed of IPA and saturated PC, one of the most abundant phospholipids. The IPA systems were the alkyltrimethylammonium-alkylsulfate series that are composed of commonly used amphiphiles. In our earlier works, we utilized MD simulations to examine the effects of alkyl chain combinations and asymmetry on the biomimetic IPA membrane properties, showing good agreement with experimental results [22,31]. In this work, we further utilized extensive MD simulations to directly compare the packing characteristics, the overall bilayer structures, the bilayer–water interfaces, and the mechanical properties between the IPA and PC bilayer with various alkyl chain lengths in either Ld or S phase. The combined results provide detailed molecular insights into the biomimetic IPA bilayers.

## 2. Results and Discussion

To investigate and compare the membrane properties of IPA and saturated PC bilayers, we started with the PC of two identical sn-1 and sn-2 chains, and their corresponding IPAs with symmetric alkyl chain combinations. The chosen IPA systems were the alkyltrimethylammonium-alkylsulfate series. For generality, we refer to the PC and IPA as DC${}_{n}$PC and C${}_{n}$TMA-C${}_{n}$S, respectively. In this work, the chosen PC lipids were dilauroyl phosphatidylcholine (DC${}_{12}$PC), dimyristoyl phosphatidylcholine (DC${}_{14}$PC), dipalmitoyl phosphatidylcholine (DC${}_{16}$PC), and distearoyl phosphatidylcholine (DC${}_{18}$PC) with the alkyl chain length varying from 12 to 18 carbons. The corresponding IPAs with alkyl chain length of 12 to 18 carbons were dodecyltrimethylammonium-dodecylsulfate (C${}_{12}$TMA-C${}_{12}$S), tetradecyltrimthylammonium-tetradecylsulfate (C${}_{14}$TMA-C${}_{14}$S), hexadecyl- trimethylammonium-hexadecylsulfate (C${}_{16}$TMA-C${}_{16}$S), and octadecyltrimthylammonium-octadecylsulfate (C${}_{18}$TMA-C${}_{18}$S), respectively. Figure 1 displays the molecular structures of DC${}_{16}$PC and C${}_{16}$TMA-C${}_{16}$S corresponding to alkyl chain length of *n* = 16.

### 2.1. Bilayer Phases

**Figure 2:**
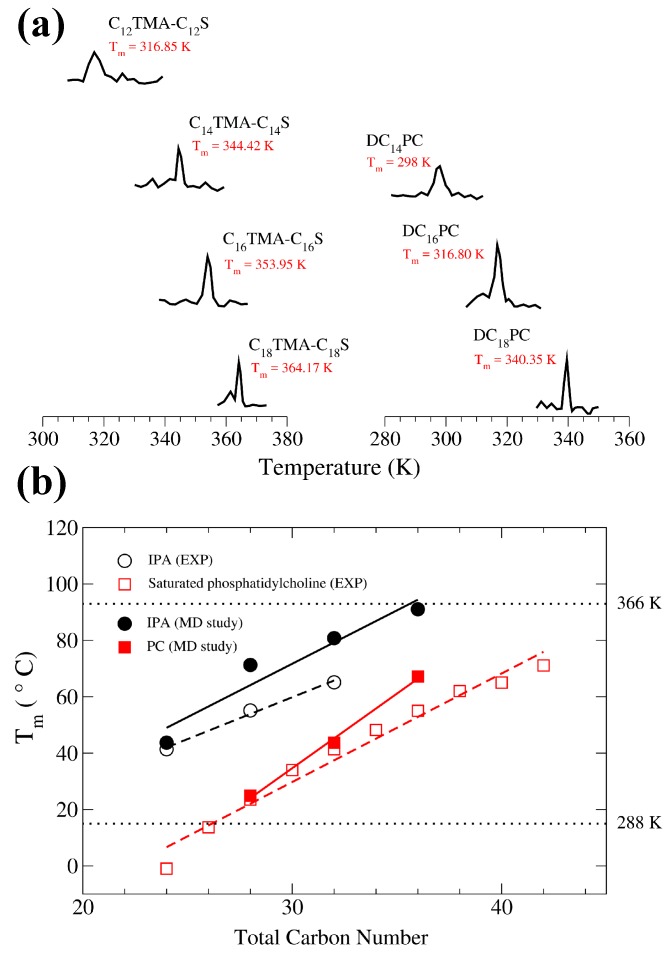
(**a**) Heat capacity profiles for DC${}_{n}$PC and C${}_{n}$TMA-C${}_{n}$S IPA bilayers with various alkyl chain length. T${}_{m}$ values denote the maximum peak positions; (**b**) T${}_{m}$ versus alkyl chain length plots for DC${}_{n}$PC and C${}_{n}$TMA-C${}_{n}$S IPA bilayers from MD (molecular dynamics) simulations (solid points) compared with experimental data (hollow points) [26,27,28].

To evaluate the main phase transition temperatures T${}_{m}$ for PC and biomimetic IPA bilayers, we performed replica-exchange molecular dynamics (REMD) simulations with 18 replicas spanning a wide temperature range as listed in Tables S1 and S2 in the supplementary material [33,34]. The heat capacity for replica *i* can be determined by:

(1) $$\begin{array}{c} C_{p}\left(T_{i}\right)={\langle \frac{dH_{i}}{dT}\rangle }_{P}=\frac{\langle H_{i}^{2}\rangle -{\langle H_{i}\rangle }^{2}}{k_{B}T_{i}^{2}}, \end{array}$$

where $k_{B}$ is the Boltzmann constant, and $T_{i}$ and $H_{i}$ are the simulated temperature and the system enthalpy for *i*th replica, respectively [35]. The resulting heat capacity-temperature profiles are shown in Figure 2. For both PC and IPA bilayers, T${}_{m}$ increases with the total alkyl chain length, consistent with experimental data [26,27]. Furthermore, IPA bilayers exhibit higher T${}_{m}$ values than the corresponding PC systems, which also agree with the results by Lee et al. [27]. Note, however, the T${}_{m}$ generated from simulations are higher than the reported experimental values, and the deviation increases with the alkyl chain length. This might be a result of the force field parameters [36,37]. For the comparison of PC and IPA membranes in S phase, we selected 288 K as the target temperature as labeled in Figure 2, lower than all tested systems excepted for DC${}_{12}$PC with T${}_{m}$ below 273 K. For MD studies of PC and IPA bilayers in Ld phase, we chose the simulation temperature of 366 K, higher than T${}_{m}$ for all systems. Notice that, for the high temperature of 366 K, the actual bilayer systems may be unstable in reality, especially for short alkyl chain system such as DC${}_{12}$PC and C${}_{12}$TMA-C${}_{12}$S IPA. However, from our preliminary MD simulations, using ensembles including constant surface tension and constant area can only transition the long alkyl chain systems with *n* >14 from the gel phase into the ripple phase with staggered alkyl chains between two leaflets and cannot give Ld phase bilayers. Alternatively, the fictitious Ld bilayers properties for long alkyl chain systems at low temperature may be predicted via extrapolation from high temperature data where the structural and mechanical properties of the bilayer under the same phase exhibit linear correlations with temperature, as revealed from previous simulation and experimental studies [38,39,40]. Considering the potential difference between the linear scaling factors of PC and IPA systems, here we chose MD simulations at high temperature of *T* = 366 K to directly compare the PC and IPA bilayers in Ld phase and examine the effects of alkyl chain length on both systems.

### 2.2. Bilayer Structures

**Figure 3:**
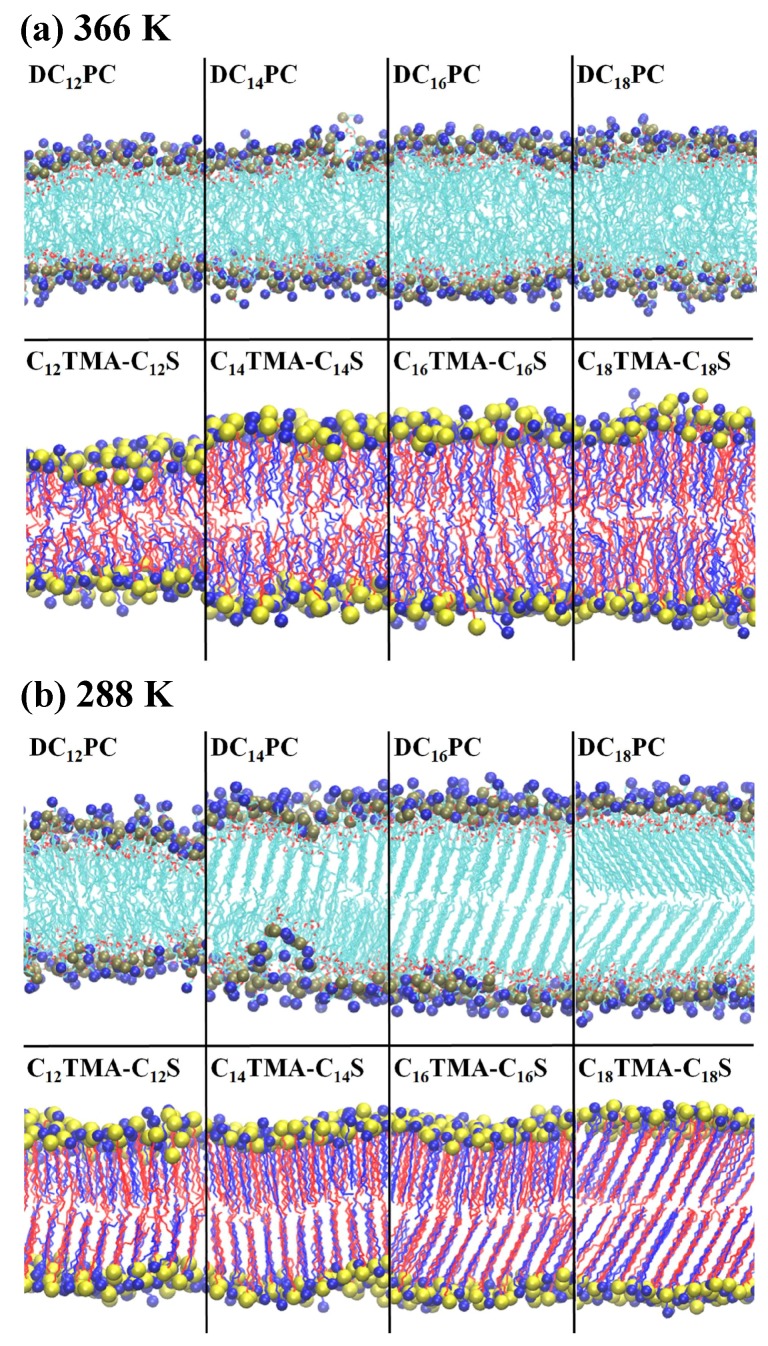
Simulation snapshots for PC and IPC systems with various alkyl chain length at (**a**) 366 and (**b**) 288 K. The atoms within the hydrophilic groups of IPA or PC are colored as follows: nitrogen is in blue, sulfur in yellow, and phosphor in tan. The alkyl chains from the cationic and anionic amphiphiles are colored in blue and red, respectively, to distinguish the two IPA components, whereas the PC alkyl chains are colored in cyan. Water is not shown for clarity. Figures were generated with VMD [32].

Series of MD simulations of PC and IPA bilayers were conducted to compare their structural and mechanical properties at molecular level. Figure 3 shows the representative configurations for the PC and IPA bilayers from equilibrium MD trajectories. At 366 K, all the tested PC and IPA bilayers exhibit disordered alkyl chain characteristics, indicating all systems are in Ld phase. At *T* = 288 K, all the tested IPA bilayers are in S phase with tilted and ordered alkyl chain packing, consistent with the reported T${}_{m}$ data of IPA [27]. However, the phase behaviors for PC bilayers at 288 K are more complicated than the biomimetic IPA systems. DC${}_{16}$PC and DC${}_{18}$PC bilayers with experimental T${}_{m}$ of 314 and 328 K, respectively, are indeed in S phase with high alkyl chain orders [26,28]. Meanwhile, DC${}_{14}$PC bilayer exhibits staggered alkyl chain packing between the two leaflets at 288 K, suggesting the system is in the ripple phase. This is consistent with the experimental T${}_{m}$ of 297 K and the pre-transition temperature T${}_{p}$ of 283 K for DC${}_{14}$PC [41,42]. In contrast, DC${}_{12}$PC bilayer exhibits low alkyl chain ordering at 288 K. This indicates the system is in Ld phase at 288 K, consistent with experimental T${}_{m}$ of 271 K [26,28].

Table 1 summarizes the molecular areas, membrane thicknesses, and the alkyl chain tilt angles for PC and IPA bilayers at 366 K. The molecular area, $\langle A_{mol}\rangle$, reflects the molecular lateral packing characteristics within the bilayer and is calculated as

(2) $$\begin{array}{c} \langle A_{mol}\rangle =\frac{\langle A_{sys}\rangle }{N_{mol}}\; \end{array}$$

where $N_{mol}$ = 64 is the number of PC molecules (or IPA complexes) per leaflet, and $\langle A_{sys}\rangle$ is the averaged lateral area of the membrane. The bilayer thickness, *h*, is defined from the transverse density profile as the distance between the peaks of the hydrophilic groups from the two leaflets. The molecular tilt angle is defined as the angle between the bilayer normal and the directive vector of the alkyl chains. For an IPA complex, two directive vectors are defined for the two amphiphilic components, each connected the first alkyl carbon and the second terminal alkyl carbon of the ionic amphiphile. Analogous to IPA complex, two directive vectors were assigned to the sn-1 and sn-2 chains of a PC molecule, each connecting the carbonyl carbon and the second last carbon of the alkyl chain.

As listed in Table 1, at 366 K where all systems are in Ld phase, the bilayer thicknesses for both PC and IPA systems increase with longer alkyl chain length as expected. Meanwhile, both the molecular area and the molecular tilt decrease with an increased alkyl chain. This is mainly due to the enhanced van der Waals (vdW) interactions within the hydrophobic regions. Comparing the PC and IPA bilayers, the thickness for an IPA bilayer is surprisingly greater than that for the corresponding PC system, considering the smaller hydrophilic head groups for an IPA complex than that for a PC molecule. This can be related to the smaller tilt angles within IPA membranes. Furthermore, the molecular area for an IPA bilayer is significantly smaller, suggesting more dense packing within an IPA bilayer than the corresponding PC system in Ld phase. The simulated molecular area were comparable to the experimental values with the same trends of decreased molecular area with elongated chains. Note that the molecular areas from MD were higher than the experimental data, possibly due to the force field parameters and the difference between the simulation temperature of 366 K and experimental condition of 333 K.

The molecular areas, membrane thicknesses, and the alkyl chain tilt angles for PC and IPA bilayers at 288 K are summarized in Table 2. At 288 K, where all tested IPA bilayers are in the S phase, the bilayer thickness increases with longer alkyl chains. Unlike Ld phase, the molecular area for an S phase IPA bilayer slightly increases with alkyl chain length. This is mainly due to the slightly increased tilt angle of longer alkyl chains. For PC bilayers at 288 K, the alkyl tilt also increases with alkyl chain length, consistent with experimental data [43]. This means that membrane thickness decreased as the alkyl chain length increased. Because the DC${}_{12}$PC bilayer is in Ld phase at 288 K, it exhibits a much lower membrane thickness and a greater molecular area. As for the DC${}_{14}$PC bilayer, because of the staggered alkyl chain between the two leaflets, its molecular area is slightly greater than that of DC${}_{16}$PC and DC${}_{18}$PC systems. Comparing the IPA and PC bilayer in the S phase, i.e., 16 and 18 alkyl carbons, IPA bilayers have a much smaller molecular area than the PC membranes, while the bilayer thicknesses are comparable between the two bilayer systems. We also analyzed two-dimensional radial distribution functions (2D-RDFs) between the charged groups for IPA and PC systems as shown in Figures S1 and S2 in the supplementary material. Indeed, the resulting RDF profiles for IPA systems exhibit more undulations at long range than the PC systems, i.e., >1.5 nm for Ld phase and >2.2 nm for S phase, suggesting that IPA bilayers have a more orderly packed hydrophilic region. Such an effect results from the strong electrostatic attractions between the charged hydrophilic groups of the IPA complex.

To characterize the packing characteristics within the bilayer hydrophobic region, we analyzed the deuterium order parameter, $|S_{CD}|$, defined as

(3) $$\begin{array}{c} S_{CD}=\frac{1}{2}\langle 3\cos^{2}\left(\theta_{CH}\right)-1\rangle \; \end{array}$$

where $\theta_{CH}$ denotes the angle between a carbon–hydrogen (C–H) bond and the bilayer normal, and the angle bracket is the ensemble average. In general, the $|S_{CD}|$ value can be used to identify whether a lipid bilayer is the gel or liquid disordered phase: the $|S_{CD}|$ profile is typically smaller than 0.2 for a Ld phase lipid bilayer, and greater than 0.2 for an S phase lipid bilayer [46,47].

**Figure 4:**
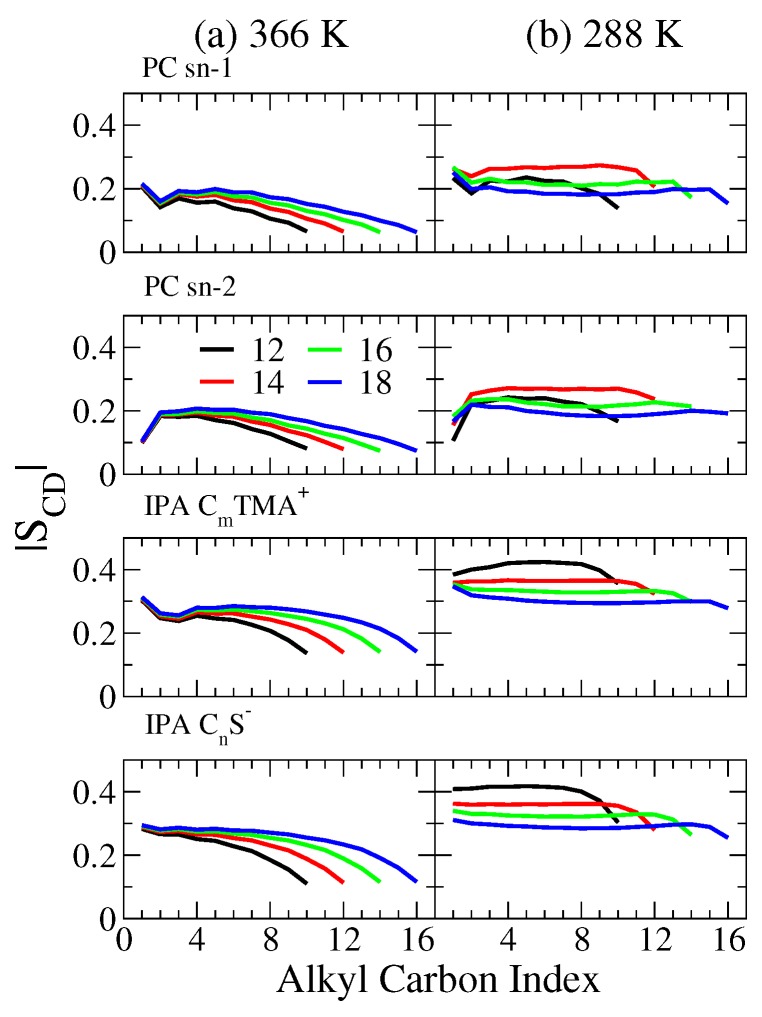
Deuterium order parameter, $|S_{CD}|$, profiles at (**a**) 366 and (**b**) 288 K for the sn-1 and sn-2 chains of PC and the alkyl chains of cationic and anionic IPA components. The color codes for the alkyl chain lengths are black, red, green, and blue for *n* = 12, 14, 16, and 18, respectively.

As shown in Figure 4, at 366 K, all the PC bilayers have $|S_{CD}|$ values smaller than 0.2, indicating that all PC systems are indeed in Ld phase. The $|S_{CD}|$ profiles for Ld phase IPA bilayers, however, are in the range of 0.1 and 0.3, higher than the corresponding PC systems. For both bilayer systems, the $|S_{CD}|$ value increases with the alkyl chain length, suggesting a slightly enhanced alkyl chain ordering due to increased vdW interactions within the hydrophobic region. At 288 K, the $|S_{CD}|$ profiles for PC bilayers fall between 0.15 and 0.3, where the ones for IPA systems are higher than 0.3. Note that the $|S_{CD}|$ plateau value for the S phase IPA bilayer decreases with longer alkyl chains. This results from greater alkyl chain tilting for a longer chain length. A similar trend is observed for PC systems at 288 K, except for DC${}_{12}$PC, which remains in Ld phase.

**Figure 5:**
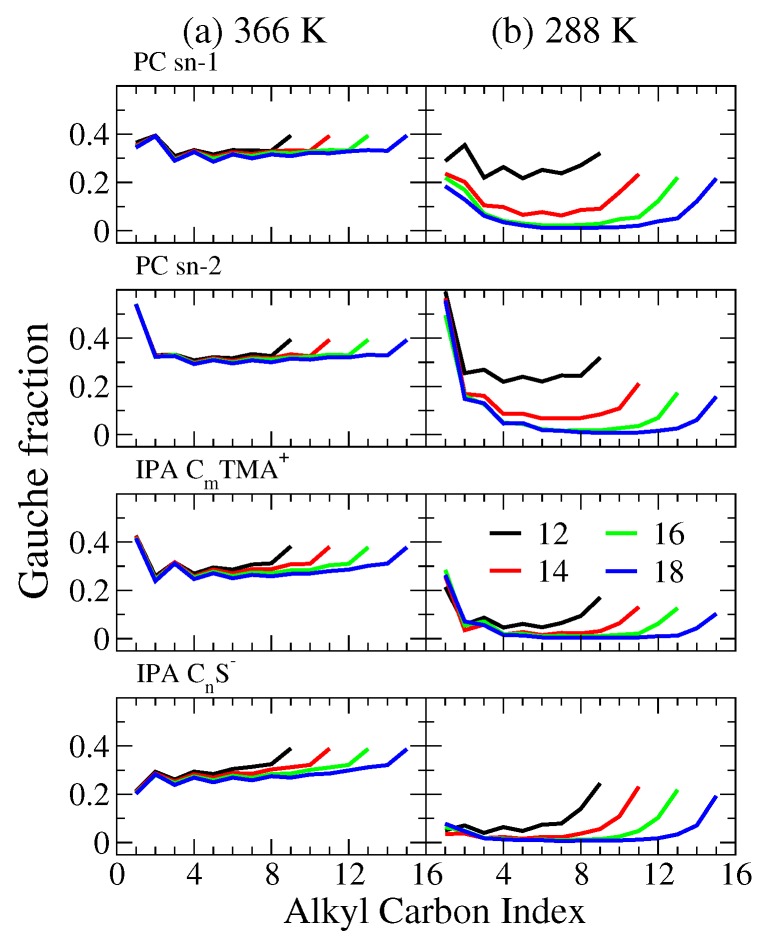
Gauche fraction profiles along the alkyl chains at (**a**) 366 and (**b**) 288 K for the sn-1 and sn-2 chains of PC and the alkyl chains of cationic and anionic IPA components. The color codes for the alkyl chain lengths are black, red, green, and blue for *n* = 12, 14, 16, and 18, respectively.

To further characterize the alkyl chain conformation, we analyzed the fraction of gauche conformers along alkyl chains as shown in Figure 5. Here, a gauche conformation is defined when the alkyl dihedral angle is in the range from −120${}^{\circ }$ to 120${}^{\circ }$. At 366 K, the gauche fractions slightly decrease with alkyl chain lengths for both PC and IPA bilayer systems, where the reduction is more pronounced for IPA systems. This indicates a more orderly alkyl chain packing resulting from the increased vdW interaction for the elongated alkyl chain. At 288 K, the gauche fractions for all IPA bilayers are near zero except for the first and the last two dihedrals, indicating the systems are in S phase where all alkyl chains are in fully extended conformations and tightly packed. For PC systems, since DC${}_{12}$PC remains in the Ld phase at 288 K, its gauche fraction profile is similar to that at 366 K. For DC${}_{14}$PC, which is in ripple phase at 288 K, its gauche fraction profile is around 0.1, between the values of 0.2 for Ld phase and nearly zero for S phase. This suggests a moderate alkyl chain packing order in the ripple phase. For the S phase, DC${}_{16}$PC and DC${}_{18}$PC systems at 288 K, the gauche fraction values are around zero at the middle alkyl chain segments, indicating densely packed alkyl chain conformations.

The combined results indicate that, for both Ld and S phase bilayers, IPA bilayers exhibit a lower gauche fraction and higher $|S_{\mathrm{CD}}|$ values than the PC ones. This suggests that alkyl chains are more densely and orderly packed in the IPA bilayers than in the PC bilayers. This can also be correlated with the smaller lateral areas and the increased thicknesses of the IPA bilayers. Such structural difference can influence the membrane–water interactions and the membrane mechanical properties, which will be discussed in the following sections.

### 2.3. Mechanical Properties

**Figure 6:**
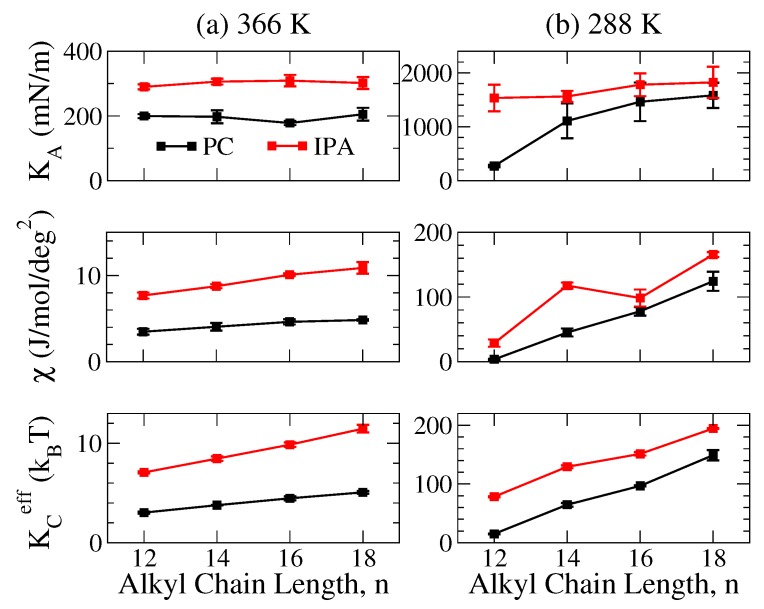
Three mechanical moduli for PC (black) and IPA (red) bilayers with various chain length, *n*, at (**a**) 366 and (**b**) 288 K: the area compressibility modulus $K_{A}$ (top panel), molecular tilt modulus χ (middle panel), and effective bending rigidity $K_{C}^{eff}$ (bottom panel). The standard deviations were evaluated from three independent MD runs.

To characterize and compare the mechanical properties of PC and the corresponding IPA bilayers, we evaluated three different membrane mechanical moduli, i.e., the area compressibility modulus $K_{A}$, the molecular tilt modulus χ, and the effective bending rigidity $K_{C}^{eff}$, for both systems as illustrated in Figure 6.

The area compressibility modulus $K_{A}$ characterizes the bilayer resistance against the membrane lateral deformation. $K_{A}$ can be evaluated from MD trajectories via the linear response theory [48,49]:

(4) $$\begin{array}{c} K_{A}=\frac{k_{B}T\langle A_{mol}\rangle }{N\langle \delta A_{mol}^{2}\rangle }\;, \end{array}$$

where $k_{B}$ is the Boltzmann constant, *T* is the simulated temperature, $\langle A_{mol}\rangle$ is the average lateral area per molecule, and *N* is the number of the molecule per leaflet, which is 64 molecules in our simulation, and $\langle \delta A_{mol}^{2}\rangle$ is the variance of $A_{mol}$.

As shown in Figure 6, both PC and IPA membranes in S phase have $K_{A}$ values one order of magnitude greater than that of Ld phase membranes. Within an S phase bilayer, the alkyl chains are packed in an ordered manner to maximize vdW interactions, leading to a greater $K_{A}$ compared to the Ld phase bilayer with disordered alkyl chains. Furthermore, for PC or IPA bilayers of the same phase conditions, i.e., S or Ld phases, $K_{A}$ values have limited variation with the alkyl chain lengths. This indicates that varying the alkyl chain length only has minor effects on membrane lateral deformation for both PC or IPA bilayers.

The molecular tilt modulus, χ, describes the required energy for changing the alkyl chain tilt within the bilayer. In this work, χ was obtained via the quadratic fitting to the potential of mean force (PMF) profile of molecular tilting F(θ) that was evaluated from the Boltzmann inversion of the normalized tilt angle distribution P(θ) [50,51]:

(5) $$\begin{array}{c} F\left(\theta \right)=-k_{B}Tln\left[\frac{P(\theta )}{\sin(\theta )}\right]=F\left(\theta_{0}\right)+\frac{\chi }{2}{\left(\theta -\theta_{0}\right)}^{2}\;, \end{array}$$

where θ denotes the molecular tilt of the alkyl chain, sin(θ) is the Jacobian factor for normalization, and $F(\theta_{0})$ is the free energy minimum at the equilibrium tilt angle $\theta_{0}$.

As revealed in Figure 6, the χ values for an S phase PC or IPA bilayer is one order of magnitude greater than the corresponding Ld phase system. This corresponds to the higher alkyl chain ordering within the S phase membrane. In addition, elongating the alkyl chain can significantly increase the χ value for both S phase PC and IPA bilayers but only causes gradual enhancements of χ for Ld phase systems. This indicates that the alkyl chain disordering compensates the enhancing effect on χ resulting from increasing the alkyl chain length.

The effective bending rigidity $K_{C}^{eff}$ characterizes the bilayer resistance against the bending deformation. In this work, $K_{C}^{eff}$ was evaluated from the molecular splay modulus $\chi_{ij}$, as recently proposed by Khelashvili et al. [51,52]. $\chi_{ij}$ measures the energy cost for splaying two molecules and can be estimated from the splay angle PMF $F(\theta_{ij})$ converted from Boltzmann inverting the splay angle distribution $P(\theta_{ij})$ followed by a quadratic fit, similar to the molecular tilt modulus [51,52]:

(6) $$\begin{array}{c} F\left(\theta_{ij}\right)=-k_{B}Tln\left[\frac{P(\theta_{ij})}{\sin(\theta_{ij})}\right]=F_{0}+\frac{\chi_{ij}}{2}{\left(\theta_{ij}\right)}^{2}\;. \end{array}$$

Here, the splay angle $\theta_{ij}$ denotes the angle between two molecular directive vectors *i* and *j*, as defined previously. $P(\theta_{ij})$ was collected under the conditions that (1) the *i* and *j* molecules were separated less than 10 Å and that (2) at least one of the molecule tilts was in the range of Θ±σ, where Θ is the most probable tilt angle and σ is 5${}^{\circ }$ for S phase systems and 10${}^{\circ }$ for the Ld phase system, respectively. The effective bending rigidity $K_{C}^{eff}$ was then evaluated by the weighed sum of splay modulus $\chi_{ij}$ for all possible molecular *i*–*j* pair types [51,52]:

(7) $$\begin{array}{c} \frac{1}{K_{C}^{eff}}=\frac{1}{\sum \varphi_{ij}}\sum \frac{\varphi_{ij}}{\chi_{ij}} \end{array}$$

where $\varphi_{ij}$ denotes the number of neighboring pairs.

From Figure 6, both PC and IPA bilayers in S phase exhibit $K_{C}^{eff}$ values that are one to two orders of magnitude higher than that of the Ld phase systems. For an S phase bilayer, the alkyl chains are well packed within the hydrophobic region, leading to a higher bending rigidity. For the S phase PC and IPA bilayers, $K_{C}^{eff}$ significantly increases as the alkyl chain elongated, mainly due to the higher alkyl chain ordering caused by the increased vdW interactions for longer alkyl chains. Similar to χ, such enhancement becomes less pronounced in Ld phase membranes, suggesting that the disorder alkyl chains within the Ld phase bilayer compensate the enhanced vdW interactions resulting from increased alkyl chain length.

Comparing the two systems, IPA bilayers have all three mechanical moduli greater than that of the corresponding PC systems in either S or Ld phases, suggesting a stronger resistance of the IPA bilayer against various deformations. These results can be correlated with more orderly packing both within the hydrophilic and hydrophobic regions for the IPA bilayer. When in Ld phase, because the disorder alkyl chain conformations counterbalance the dense packing within the IPA, the mechanical strength are comparable between the two systems. For S phase systems, the higher alkyl chain orders within IPA systems, as reflected in the smaller molecular area, a higher $|S_{CD}|$ value, a lower gauche fraction, etc., results in significantly higher mechanical moduli of IPA bilayers than that of the corresponding PC membranes.

### 2.4. The Bilayer–Water Interface

In the vicinity of the bilayer–water interface, we first analyzed the number of hydrogen bonds (H-bonds) formed between the water and the hydrophilic groups of the IPA and PC bilayer systems, as summarized in Table 1. Both PC and IPA bilayers can form more H-bonds with water in Ld phase at 366 K than in S phase at 288 K, mainly due to a larger molecular area, allowing more contact with water molecules. The only exception is the DC${}_{12}$PC system that is in Ld phase at both temperatures, and the number of H-bonds is reduced at 366 K due to the thermal effect. Furthermore, PC bilayers form more H-bonds with water than IPA systems. Considering the molecular structure, the phosphate and glycerol esters of PC can participate in H-bonding interactions. In contrast, only the sulfate group within an IPA complex can form H-bonds with water, leading to fewer overall H-bonds. Note that the number of H-bonds does not vary with the alkyl chain length for IPA and PC bilayers in either Ld or S phases, suggesting a minor effect of alkyl chain on the bilayer–water H-bonding interactions.

**Figure 7:**
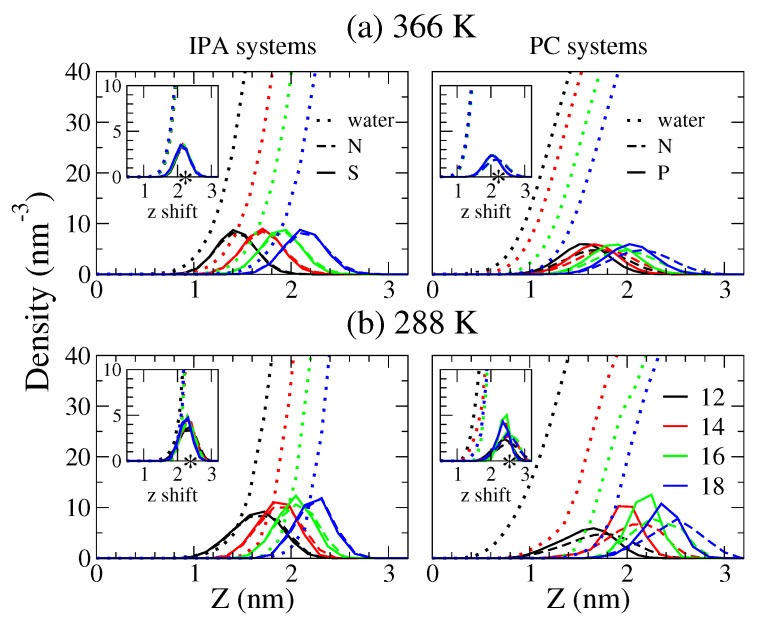
Transverse density profiles for water (dotted line), N atoms for the positively charged trimethylammonium of IPA and choline of PC (dashed line), and the negatively charged groups of S atoms for IPA sulfate and P atoms for PC phosphate (solid line) at (**a**) 366 and (**b**) 288 K, respectively. The color codes for the alkyl chain lengths are black, red, green, and blue for *n* = 12, 14, 16, and 18, respectively. The insets display the density profiles shifted with respect to the N atom distributions of the systems with the alkyl chain length of *n* = 18. The most probable positions for N are marked with star symbols (*). The densities for the hydrophilic groups are amplified 5 times for clarity.

Figure 7 displays the transverse density profiles of water and the hydrophilic groups of IPA and PC bilayers. At 366 K, all the PC and IPA membranes are in Ld phase where water can permeate approximately 1 nm below the hydrophilic groups in both systems. At 288 K, the IPA systems are in S phase, restricting water above the hydrophilic region. In contrast, water can penetrate deeper into the PC bilayers at 288 K, not only for Ld phase DC${}_{12}$PC and ripple phase DC${}_{14}$PC but also for S phase DC${}_{16}$PC and DC${}_{18}$PC. This indicates the smaller molecular area for IPA systems that can effectively block the water permeations. Note that, for the IPA systems in either Ld or S phase, the N atoms of the trimethylammonium are aligned with the S atoms of the sulfate due to the electrostatic interactions between the charged groups. For the PC systems, the N atoms of the choline are positioned above the P atoms of the phosphate, due to the intrinsic molecular structure of PC as illustrated in Figure 1.

The insets of Figure 7 plot the density profiles for PC and IPA systems with various alkyl chain lengths shifted according to the nitrogen distributions for the systems of the longest alkyl tails, i.e., DC${}_{18}$PC and C${}_{18}$TMA-C${}_{18}$S IPA, respectively. Interestingly, except for DC${}_{12}$PC and DC${}_{14}$PC at 288 K that are in a different phase condition, the water density profiles are well matched for both PC and IPA bilayers with different alkyl chain length in S and Ld phase. This suggests that the alkyl chain length has little effect on the hydrophilic region for either IPA or PC bilayers in Ld or S phase.

The hydrophilic composition can also affect water orientations, which are important factors for the hydrodynamic characteristics of the bilayer. The water orientation can be characterized via the angle α between the electric dipole and the bilayer normal. Here, we analyzed the normalized water orientational angle distributions at various transverse positions *z*. Since the alkyl chain length has little effect on the water transverse distribution near the hydrophobic region for either the IPA or PC bilayer as illustrated in the insets of Figure 7, we only plotted the representative orientational data of the DC${}_{18}$PC and C${}_{18}$TMA-C${}_{18}$S IPA bilayers at 288 and 366 K.

**Figure 8:**
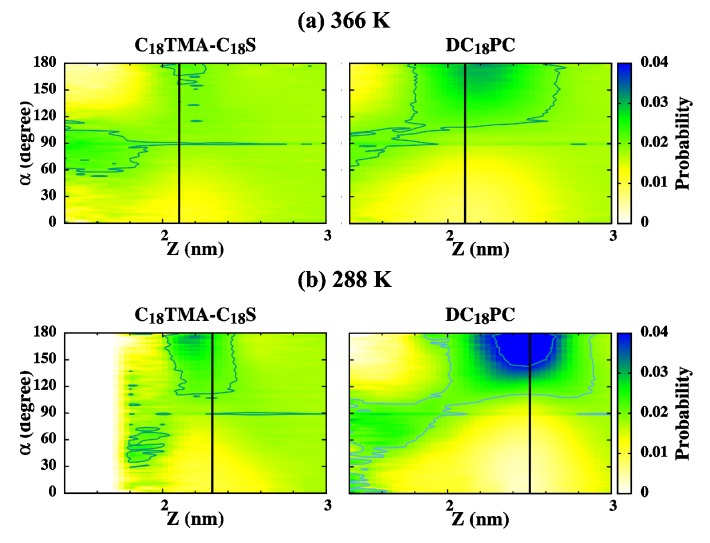
The water orientational angle distributions at various transverse positions, *z*, for the C${}_{18}$TMA-C${}_{18}$S IPA and DC${}_{18}$PC bilayer at (**a**) 366 and (**b**) 288 K. The black lines denote the most probable positions for the central N atoms of the positively charged trimethylammonium of IPA and choline of PC as marked with star symbols in Figure 7.

As shown in Figure 8, for the DC${}_{18}$PC and C${}_{18}$TMA-C${}_{18}$S IPA bilayers in Ld phase at 366 K, water molecules have large orientational angles of >90${}^{\circ }$, with H atoms facing the membrane when they approach the bilayer–water interface. As the water permeates toward the bilayer interior, the water orientational angles gradually reduce to <90${}^{\circ }$, i.e., with O atoms facing the bilayer center. Such orientational transition is mainly due to the H-bonding interactions between the H atoms of water and the sulfate groups of IPA and the ester groups of PC, respectively. The water orientational change are more pronounced for the S phase bilayers at 288 K. According to the transverse density profiles, the position distributions of the bilayer hydrophilic charged groups are narrower for the S phase bilayers at 288 K, leading to more well-defined water orientational preferences for the S phase bilayer systems. Note that, for IPA systems, the negatively charged sulfate is aligned with the ammonium; for PC bilayers, the phosphate are located below the choline group. The water dipole is affected by the vertically oriented P–N dipole within the hydrophilic groups of the PC molecules. Thus, at the bilayer–water interface, the water orientation has a more prominent preference of >90${}^{\circ }$ for the PC system compared with the IPA system.

**Figure 9:**
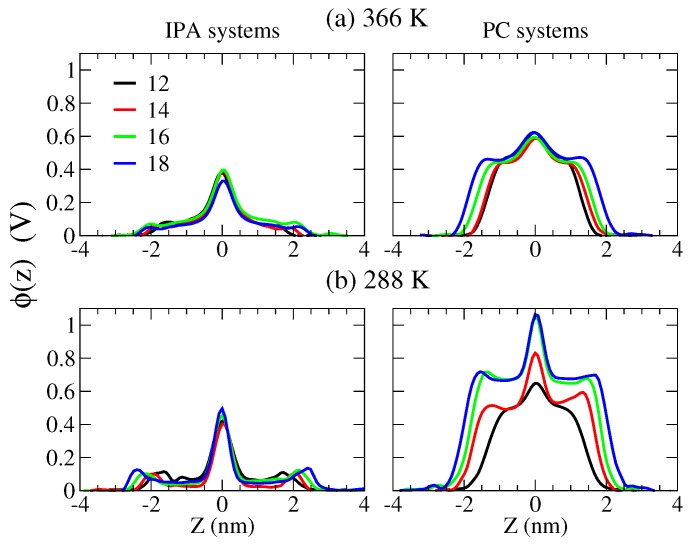
Electrostatic potential profiles along the bilayer normal at (**a**) 366 and (**b**) 288 K for C${}_{n}$TMA-C${}_{n}$S IPA and DC${}_{n}$PC bilayers. The color codes for the alkyl chain lengths are black, red, green, and blue for *n* = 12, 14, 16, and 18, respectively.

The different alignment of charged groups for PC and IPA also results in the variations in the electrostatic potentials for both membranes. The electrostatic potential was computed based on the Poisson’s equation via the double integration of the charge density profile [53,54]:

(8) $$\begin{array}{c} \phi \left(z\right)-\phi \left(0\right)=\frac{-1}{\epsilon_{0}}\int_{0}^{z}dz^{\prime }\int_{0}^{z^{\prime }}\rho \left(z^{\prime \prime }\right)dz^{\prime \prime } \end{array}$$

where *z* is the distance with respect to the bilayer center of mass along the bilayer normal, $\epsilon_{0}$ denotes the vacuum permittivity, ρ(z) is the charge density at *z*, and ϕ(0) is the electrostatic potential as bilayer center. As shown in Figure 9, the overall electrostatic potential varies only in the *z* range but little in magnitude as the alkyl chain elongates for both bilayer systems at either temperature. The potentials for S phase systems are higher than the corresponding Ld phase membranes, due to the narrower density distributions in the S phase. More importantly, the potentials for IPA systems are significantly smaller than the PC bilayers. The lateral charge alignment within the IPA complex, compared with the transverse charge distributions of a PC, results in reduced membrane potentials.

To characterize the dynamic properties of water at the bilayer–water interface, we calculated the water residence time τ on the bilayer surface from the correlation function R(t) [55,56,57]:

(9) $$\begin{array}{c} \tau =\int_{0}^{\infty }\langle R(t)\rangle dt=\int_{0}^{\infty }\langle \frac{1}{N_{0}}\sum P_{i}\left(t\right)\rangle dt. \end{array}$$

$N_{0}$ is the number of water within 0.45 nm of the phosphorus of PC and sulfur of IPA at time 0, which corresponds to the first solvation shell. $P_{i}\left(t\right)$ is the Heaviside function, which is 1 if the *i*th molecule is found in the solvation shell at both time 0 and *t*, and 0 otherwise. The correlation function $\langle R(t)\rangle$ was fitted to a bi-exponential functions of $A_{0}exp\left(-t/\tau_{0}\right)+A_{1}exp\left(-t/\tau_{1}\right)$ for the convergence of integration [56,57].

**Figure 10:**
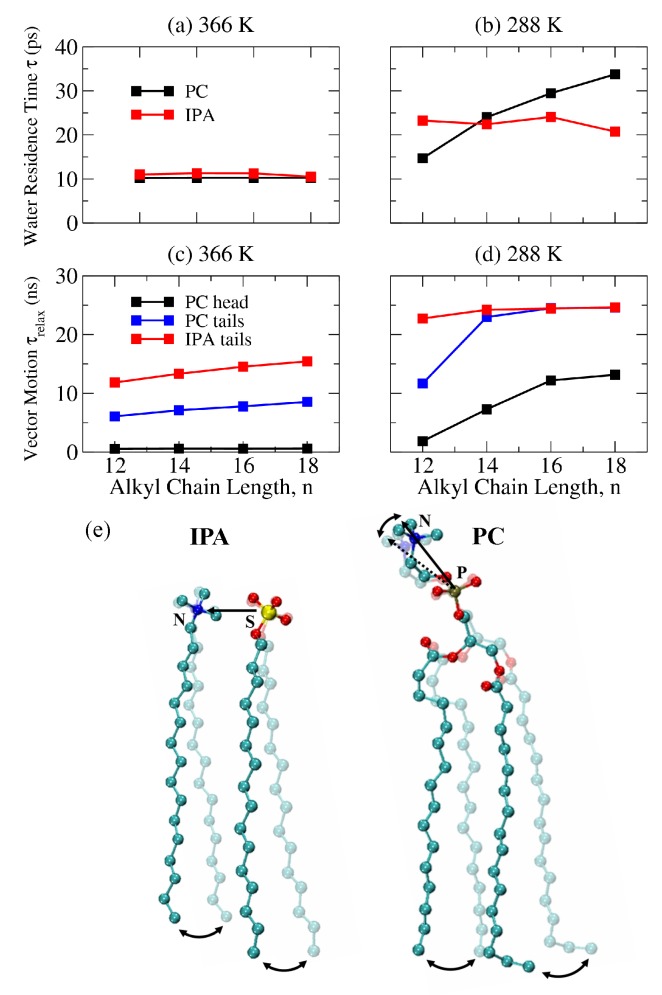
Water residence time τ at (**a**) 366 and (**b**) 288 K for DC${}_{n}$PC and C${}_{n}$TMA-C${}_{n}$S IPA bilayers with various alkyl chain lengths. The orientational relaxation time $\tau_{relax}$ at (**c**) 366 and (**d**) 288 K for the hydrophilic heads (P–N vectors) and the hydrophilic tails of PC and the alkyl chains of IPA. (**e**) Illustrations of the couplings between the alkyl tilting and the dipole of hydrophilic groups for an IPA complex and a PC molecule. Atomic color codes are: nitrogen in blue, sulfur in yellow, phosphorus in brown, oxygen in red, and carbon in turquoise, respectively.

As shown in Figure 10a,b, PC and IPA bilayers at 366 K have shorter water residence time τ than those at 288 K, mainly due to the rapid motion of water at high temperature. Additionally, τ at 366 K does not vary with the alkyl chain length, indicating a limited effect of the alkyl chain length on the water dynamics on the Ld phase bilayer surface. At 288 K, τ surprisingly increases with alkyl chain length for the PC systems but remains constant for the S phase IPA bilayers of various chain lengths. To elucidate this, we calculated the relaxation time of the molecular tilting $\tau_{relax}$ for the PC and IPA bilayers via the integration of the rotational autocorrelation function C(t) of the directive vector [58,59,60,61]:

(10) $$\begin{array}{c} \tau_{relax}=\int_{0}^{\infty }\langle C(t)\rangle dt=\int_{0}^{\infty }\langle P_{2}\left(\cos\;\theta (t_{0},t_{0}+t)\right)\rangle dt. \end{array}$$

$\theta (t_{0},t_{0}+t)$ denotes the angle between the directive vector at time $t_{0}$ and $t_{0}+t$, and $P_{2}\left(x\right)=0.5\;\times \left(3x^{2}-1\right)$ is the second Legendre polynomial. $\langle C(t)\rangle$ was fitted to bi-exponential functions of $A_{0}^{\prime }exp\left(-t/\tau_{0}^{\prime }\right)+A_{1}^{\prime }exp\left(-t/\tau_{1}^{\prime }\right)$ for the convergence of integration [60,61]. Figure 10c,d illustrate the resulting $\tau_{relax}$ for alkyl tilts within PC and IPA bilayers and the P–N vectors for PC systems. For a P–N vector, $\tau_{relax}$ does not change significantly with the alkyl chain at 366 K; at 288 K, $\tau_{relax}$ increases with the alkyl chain length, similar to the water residence time τ. Note that, as shown in Figure S3, the P–N vector distributions are similar for both Ld phase and S phase PC bilayers with a slightly larger averaged tilt angle at 366 K, indicating a more parallel orientation of the P–N vector in the membrane lateral direction in the Ld phase. These results suggest that a tilted P–N orientation has minor effects on the water residence time on the membrane surface. Meanwhile, $\tau_{relax}$ for the alkyl chain has a similar trend toward the P–N vector result, suggesting that hydrophobic tilt relaxation correlates closely with hydrophilic groups for the PC bilayers. As demonstrated in Figure 10e, the longer alkyl chains lead to slower molecular tilting dynamics corresponding to larger $\tau_{relax}$ values for P–N vectors and hence longer water residence time τ values. However, the charged hydrophilic groups are not covalently linked for an IPA complex. The tilting of alkyl chains are therefore decoupled with the IPA hydrophilic regions and have little effect on the water dynamics at the bilayer–water interface for both Ld and gel phases.

## 3. Materials and Methods

Both PC and IPA bilayers can exhibit in either S or Ld phase at a particular temperature, depending on their characteristic T${}_{m}$ values. The phase condition can significantly influence the structural and mechanical properties of the bilayer. In order to compare the PC and IPA bilayers under the same phase condition, we performed two series of MD simulations at temperatures above and below T${}_{m}$. Here, we utilized replica-exchange molecular dynamics (REMD) with 18 replicas spanning a wide temperature range to evaluate T${}_{m}$ of PC and IPA bilayer systems [33,34]. The exchange of conformations in neighboring temperatures was attempted every 1000 time steps based on the Metropolis criterion [33,34]. For more details of REMD simulations and the temperature list for each bilayer system, please refer to the supplementary material. Our REMD results showed that all the tested PC and IPA bilayers were in Ld phase at 366 K; whereas at 288 K most PC and IPA membranes were under the S phase-like condition. Therefore, two sets of MD simulations at 366 and 288 K allowed direct comparisons between the PC and IPA bilayers in Ld and S phases.

Both IPA and PC molecules were described using the CHARMM36 all-atom force field [62]. The TIP3P model was applied for water [63,64]. The combination of TIP3P and CHARMM force fields have been used in various MD studies on C${}_{n}$TMA-C${}_{n}$ IPA and lipid bilayer systems [22,31,62]. All-atom MD simulations were performed using the GROMACS 5.0.4 package under the isothermal-isobaric (NPT) ensemble [65]. The temperature was maintained at 366 or 288 K via the Nose–Hoover thermostat [66,67,68]. The pressure was controlled at 1 bar using the semi-isotropic Parrinello–Rahman algorithm [69]. The Lennard–Jones potential were cut off at 1.2 nm and smoothly shifted to zero starting from 0.8 nm. The particle mesh Ewald (PME) method were applied to evaluate long-range electrostatic interactions [70,71]. Chemical bonds involving hydrogen atoms were constrained at their equilibrium length using the LINCS algorithm [72]. An integration time step of 2 fs was used to evaluate the equations of motion of atoms. System configurations were saved every 10 ps for further data analysis.

The initial IPA and PC bilayer conformations were constructed through PACKMOL and CHARMM-GUI [73,74]. Each bilayer was composed of 128 PC lipids (or IPA complexes) with 64 lipids per leaflet and solvated with 3464 water molecules, suggesting the fully hydrated bilayers with the water to lipid ratio of 27 [75]. All initial conformations were energy -minimized via the steepest descent algorithm to eliminate any bad contacts. Each system was then equilibrated at 366 K and 1 bar for 180 ns at which the bilayer was in the liquid disordered phase. The last 100 ns trajectories were used for analyzing the Ld phase bilayer properties. For an S phase bilayer, the system was further annealed from 366 to 288 K with a 2.5 K/ns cooling rate [29,30,76]. After the annealing, the system was then equilibrated for 200 ns followed by an additional 100 ns of production run for further analysis of the S phase bilayer properties. To check the system equilibration, we compared the area distributions and the alkyl chain tilt angle distributions for each bilayer system from the first and the second 50 ns periods of the 100 ns production MD run as shown in Figures S4 and S5 in the supplementary material. All the analyses were conducted with the in-house codes using the algorithms described in the main text. Three independent MD runs were conducted for each system to ensure reproducibility.

## 4. Conclusions

In this work, we employed a series of MD simulations to compare the structural and mechanical properties of bilayer systems composed of DC${}_{n}$PC lipids and biomimetic C${}_{n}$TMA-C${}_{n}$ IPA complexes. The MD simulations were conducted at two different temperatures of 288 and 366 K to investigate the bilayer systems in S and Ld phase, respectively. As the alkyl chain elongates, the bilayer thickness significantly increases for both IPA and PC bilayers in either Ld or S phase. The molecular area and the alkyl chain ordering are reduced, owing to the increased vdW interactions between the elongated alkyl chains. The molecular structures of PC and IPA only differs at the compositions of hydrophilic groups. The strong electrostatic attraction between the two IPA components results in a reduced molecular area for IPA bilayers, both in Ld and S phase. Additionally, the alkyl chains have smaller tilting angles in the IPA bilayers compared with the PC systems. These can correspond to an increased alkyl chain ordering of an IPA bilayer, in either Ld or S phase, as revealed by the $|S_{CD}|$ and gauche fraction analyses. The combined results indicate that an IPA bilayer, compared with a PC system, exhibits more densely and orderly molecular packing in both hydrophilic and hydrophobic regions.

For an IPA bilayer in which the components are more densely packed, the area compressibility modulus $K_{A}$, the molecular tilt modulus χ, and the effective bending rigidity $K_{C}^{eff}$ are greater than the corresponding PC bilayer. Meanwhile, the mechanical strengths for both PC and IPA bilayers can also be enhanced by elongating the alkyl chain that increases vdW interactions within the membrane. These mechanical enhancing effects can be compensated by the alkyl chain disordering and are less pronounced in the Ld phase systems. Therefore, the mechanical moduli for the Ld phase PC and IPA bilayers are more comparable.

The difference between the hydrophilic groups of PC and IPA also impacts the bilayer–water interfacial properties. A PC bilayer in either S or Ld phase forms more H-bonds with water than the IPA membrane, due to the more H-bond forming groups within the PC hydrophilic region. Furthermore, an IPA membrane exhibit greater resistance against water penetration due to denser molecular packing. The water orientation is also affected by the charged groups within the bilayer hydrophilic region. For a PC bilayer, water have more distinct orientational preference at the bilayer–water interface, resulting from more H-bonding interactions. The intrinsic dipole within the PC hydrophilic head further leads to a greater membrane potential than the IPA bilayer. The alkyl chain elongation does not influence the bilayer–water H-bonding and the water transverse density profiles for both PC and the IPA bilayer in either S or Ld phase. This indicates that the alkyl chain length variation has minor effects on the bilayer–water interface for both PC and IPA systems. Note that the non-covalently linked hydrophilic groups of an IPA complex results in the decoupling of alkyl chain tilting relaxation and the surface water retention, implying different hydrodynamic properties between PC and biomimetic IPA bilayers.

Through the comparison of the similarities and intrinsic differences between the PC and IPA bilayers at the molecular level, the combined MD results illustrate the importance of hydrophilic compositions on the modulation of bilayer properties. The provided molecular insights can serve as designing guidelines of novel biomimetic membranes for various applications.

## Figures and Tables

**Table 1:** The average molecular area $\langle A_{mol}\rangle$, experimental value of area per lipid $A_{exp}$, membrane thickness 〈h〉, molecular tilt angle 〈θ〉, and number of H-bonds with water $\langle N_{Hbond}\rangle$ for DC${}_{n}$PC and C${}_{n}$TMA-C${}_{n}$S IPA bilayers at 366 K.

System		$\langle A_{\mathrm{mol}}\rangle$ ^a^	$A_{\exp}$ ^b^	〈h〉 ^c^	〈θ〉	$\langle N_{\mathrm{Hbond}}\rangle$
	DC${}_{12}$PC	0.690 ± 0.017	0.659	2.36 ± 0.07	35.61 ± 19.10	6.34 ± 0.17
PC	DC${}_{14}$PC	0.684 ± 0.017	0.657	2.64 ± 0.05	33.55 ± 18.43	6.29 ± 0.17
	DC${}_{16}$PC	0.679 ± 0.017	0.650	3.08 ± 0.07	32.44 ± 18.15	6.31 ± 0.16
	DC${}_{18}$PC	0.617 ± 0.016	0.638	3.37 ± 0.08	30.89 ± 17.73	6.27 ± 0.17
	C${}_{12}$TMA-C${}_{12}$S	0.578 ± 0.013	-	2.69 ± 0.05	24.33 ± 14.35	3.41 ± 0.13
IPA	C${}_{14}$TMA-C${}_{14}$S	0.569 ± 0.012	-	3.28 ± 0.04	22.47 ± 13.56	3.37 ± 0.12
	C${}_{16}$TMA-C${}_{16}$S	0.561 ± 0.012	-	3.60 ± 0.03	20.81 ± 12.78	3.34 ± 0.12
	C${}_{18}$TMA-C${}_{18}$S	0.556 ± 0.012	-	3.88 ± 0.04	19.43 ± 12.32	3.31 ± 0.12

^a^ nm${}^{2}$ per molecule; ^b^ measured at 333 K [38]; ^c^ nm; standard deviations for 〈h〉 were evaluated from three independent MD runs.

**Table 2:** The average molecular area $\langle A_{mol}\rangle$, experimental value of area per lipid $A_{exp}$, membrane thickness 〈h〉, molecular tilt angle 〈θ〉, and the number of H-bonds with water $\langle N_{Hbond}\rangle$ for DC${}_{n}$PC and C${}_{n}$TMA-C${}_{n}$S IPA bilayers at 288 K.

System		$\langle A_{\mathrm{mol}}\rangle$ ^a^	$A_{\exp}$	〈h〉 ^b^	〈θ〉	$\langle N_{\mathrm{Hbond}}\rangle$
	DC${}_{12}$PC	0.606 ± 0.013	-	2.40 ± 0.05	29.46 ± 17.84	7.03 ± 0.16
PC	DC${}_{14}$PC	0.511 ± 0.008	0.472 ^c^	3.17 ± 0.03	29.27 ± 10.14	6.20 ± 0.16
	DC${}_{16}$PC	0.493 ± 0.005	0.473 ^d^	3.42 ± 0.04	35.01 ± 6.05	5.77 ± 0.15
	DC${}_{18}$PC	0.501 ± 0.004	0.473 ^d^	3.89 ± 0.02	37.13 ± 5.30	5.74 ± 0.15
	C${}_{12}$TMA-C${}_{12}$S	0.422 ± 0.005	-	3.15 ± 0.02	13.32 ± 8.00	2.54 ± 0.08
IPA	C${}_{14}$TMA-C${}_{14}$S	0.426 ± 0.005	-	3.64 ± 0.05	21.57 ± 5.83	2.48 ± 0.08
	C${}_{16}$TMA-C${}_{16}$S	0.431 ± 0.004	-	4.10 ± 0.06	24.25 ± 5.32	2.60 ± 0.09
	C${}_{18}$TMA-C${}_{18}$S	0.441 ± 0.004	-	4.28 ± 0.08	28.90 ± 5.24	2.62 ± 0.08

^a^ nm${}^{2}$ per molecule; ^b^ nm; standard deviations for 〈h〉 were evaluated from three independent MD runs; ^c^ measured at 283 K [44]; ^d^ measured at 298 K [45].

## Supplementary Materials

The following are available online at http://www.mdpi.com/1422-0067/19/6/1552/s1.

## Abbreviations

IPA	ion pair amphiphile
PC	phosphatidylcholine
MD	molecular dynamics
REMD	replica-exchange molecular dynamics
DC${}_{12}$PC	dilauroyl phosphatidylcholine, DLPC
DC${}_{14}$PC	dimyristoyl phosphatidylcholine, DMPC
DC${}_{16}$PC	dipalmitoyl phosphatidylcholine, DPPC
DC${}_{18}$PC	distearoyl phosphatidylcholine, DSPC
C${}_{12}$TMA-C${}_{12}$S	dodecyltrimethylammonium-dodecylsulfate
C${}_{14}$TMA-C${}_{14}$S	tetradecyltrimthylammonium-tetradecylsulfate
C${}_{16}$TMA-C${}_{16}$S	hexadecyltrimethylammonium-hexadecylsulfate
C${}_{18}$TMA-C${}_{18}$S	octadecyltrimthylammonium-octadecyllsulfate
