# Supplementary material for: Importance of Hydrophilic Groups on Modulating the Structural, Mechanical, and Interfacial Properties of Bilayers: A Comparative Molecular Dynamics Study of Phosphatidylcholine and Ion Pair Amphiphile Membranes
Source: Int J Mol Sci. 2018 May 23;19(6):1552. doi: 10.3390/ijms19061552 (PMC6032153; doi:10.3390/ijms19061552)
Supplement: Supplementary file 1 [file ijms-19-01552-s001.pdf]

Supplementary Material for

"Importance of Hydrophilic Groups on the Structural, Mechanical, and Interfacial Properties of Bilayers: A Comparative Molecular Dynamics Study of Phosphatidylcholine and Biomimetic Ion Pair Amphiphile Membranes"

Ching-an Tian[1] and Chi-cheng Chiu[1,*]

[1]Department of Chemical Engineering, National Cheng Kung University, Tainan 70101, Taiwan.

[*]Correspondence: ccchiu2@mail.ncku.edu.tw

PT-REMD Details

In order to improve the conformational sampling, we applied the parallel tempering replica exchange molecular dynamics (PT-REMD) and further calculated the heat capacity to temperature profile. The PT-REMD involves performing N simulations simultaneously at different temperatures of T_1, T_2, T_3..., T_N (1-2). The conformations from consecutive temperatures are exchanged constantly based on the Metropolis criterion.

$$p(i \leftrightarrow j) = min\left(1, e^{(E_i - E_j)\left(\frac{1}{k_B T_i} - \frac{1}{k_B T_j}\right)}\right)$$

where T_i and T_j are the reference temperature, k_B is Boltzmann's constant, and E_i and E_j are the instantaneous potential energies of replica i and j, respectively. Such approach allows the exchanges of configurations at high temperatures with the ones at low temperatures. In Monte Carlo simulations, PT-RE only considers the particle positions and potential energies. In MD, the momenta of all the particles in the system

should be also considered. After an exchange, the new momenta for replica i, $P^{(i')}$ should be adjusted as:

$$P^{(i')} = \sqrt{T_{before}/T_{after}}\, P^{(i)}$$

where $p^{(i)}$ is the original momenta for replica i, and T_{before} and T_{after} are the temperatures of the replica before and after the swap, respectively. The process ensures that the average kinetic energy remains equal to $\frac{3}{2}Nk_BT$.

We utilized REMD simulation to evaluate the transition temperature of PC and IPA bilayer systems. The list of replicas with various temperature range for all the tested systems are given in Table S1 and S2. All REMD simulations were carried out under NPT ensemble for 20 ns. The exchange of conformations in neighboring temperatures was attempted at every 1000 time steps based on the Metropolis criterion.

Table S1 Temperature list for REMD simulations of C_nTMA-C_nS IPA systems.

IPA	$C_{12}TMA-C_{12}S$	$C_{14}TMA-C_{14}S$	$C_{16}TMA-C_{16}S$	$C_{18}TMA-C_{18}S$
T_1	308.00	330.10	338.05	357.22
T_2	309.68	332.13	339.88	359.35
T_3	311.66	333.95	341.64	361.47
T_4	313.20	335.96	343.34	362.88
T_5	315.01	337.77	345.11	364.17
T_6	316.85	339.63	346.85	364.72
T_7	318.61	341.55	348.52	365.45
T_8	320.46	343.39	350.43	366.27
T_9	322.35	344.42	352.17	367.57
T_{10}	324.20	345.26	353.95	368.75
T_{11}	326.04	346.46	354.88	370.92
T_{12}	327.76	347.41	355.83	373.26
T_{13}	329.82	349.24	357.60	
T_{14}	331.54	351.34	359.44	
T_{15}	333.43	353.26	361.23	
T_{16}	335.15	355.23	363.12	
T_{17}	337.34	357.18	364.94	
T_{18}	339.23	359.33	367.07	

Table S2 Temperature list for REMD simulations of DC_nPC systems.

PC	$DC_{14}PC$	$DC_{16}PC$	$DC_{18}PC$
T_1	282.05	306.58	329.61
T_2	284.13	308.40	331.17
T_3	285.97	310.18	333.06
T_4	287.95	312.12	334.44
T_5	289.98	314.07	336.25
T_6	291.95	315.82	338.25
T_7	293.96	316.80	339.62
T_8	295.97	317.71	340.35
T_9	296.89	318.60	341.37
T_{10}	298.00	319.64	342.36
T_{11}	299.94	320.50	343.01
T_{12}	301.02	321.46	344.84
T_{13}	301.96	322.39	346.43
T_{14}	304.05	323.31	347.23
T_{15}	306.05	325.36	348.12
T_{16}	308.06	327.15	349.80
T_{17}	310.07	329.17	351.42
T_{18}	312.09	331.05	353.31

Two-Dimensional Radial Distribution Function (2D-RDF)

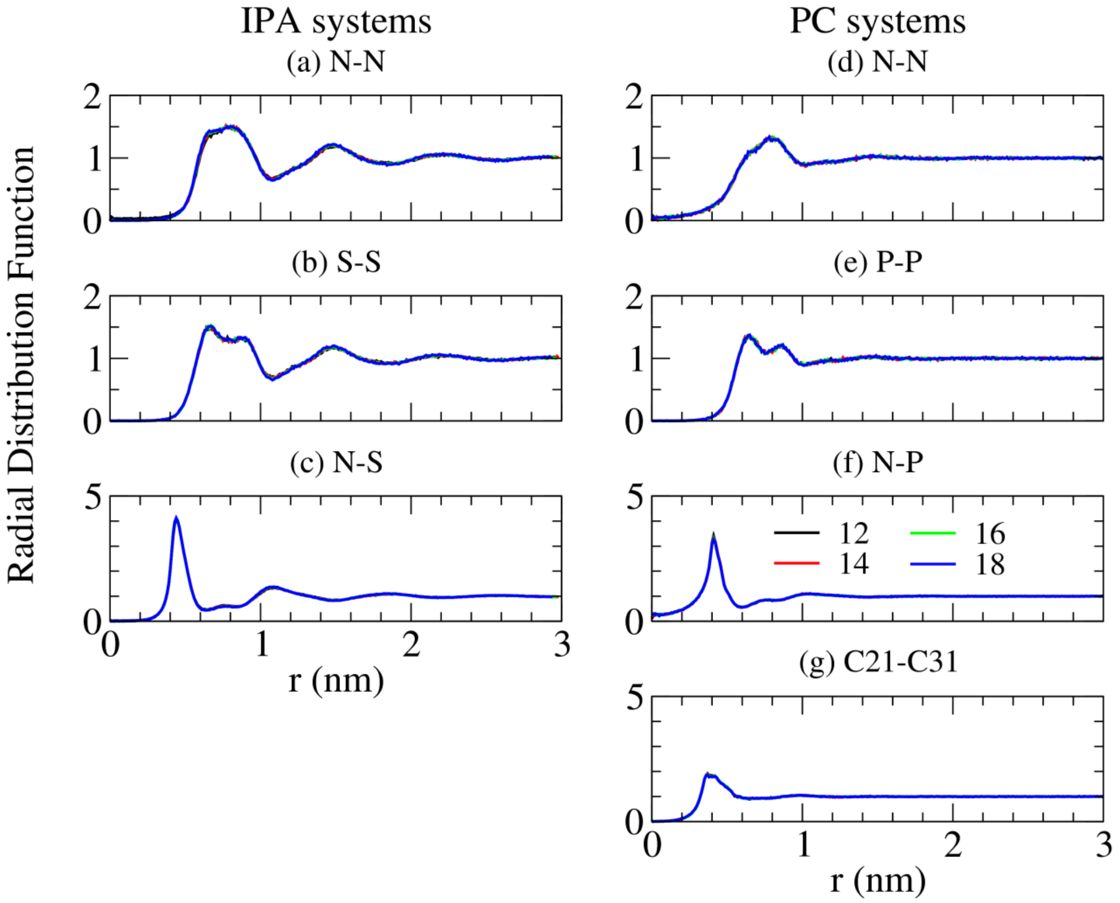

Figure S1 Two-dimensional radial distribution function of the (a) N-N pairs, (b) S-S pairs, and (c) N-S pairs for IPA bilayers and (d) N-N pairs, (e) P-P pairs, (f) N-P pairs, and (g) C21-C31 pairs for PC bilayers at 366 K.

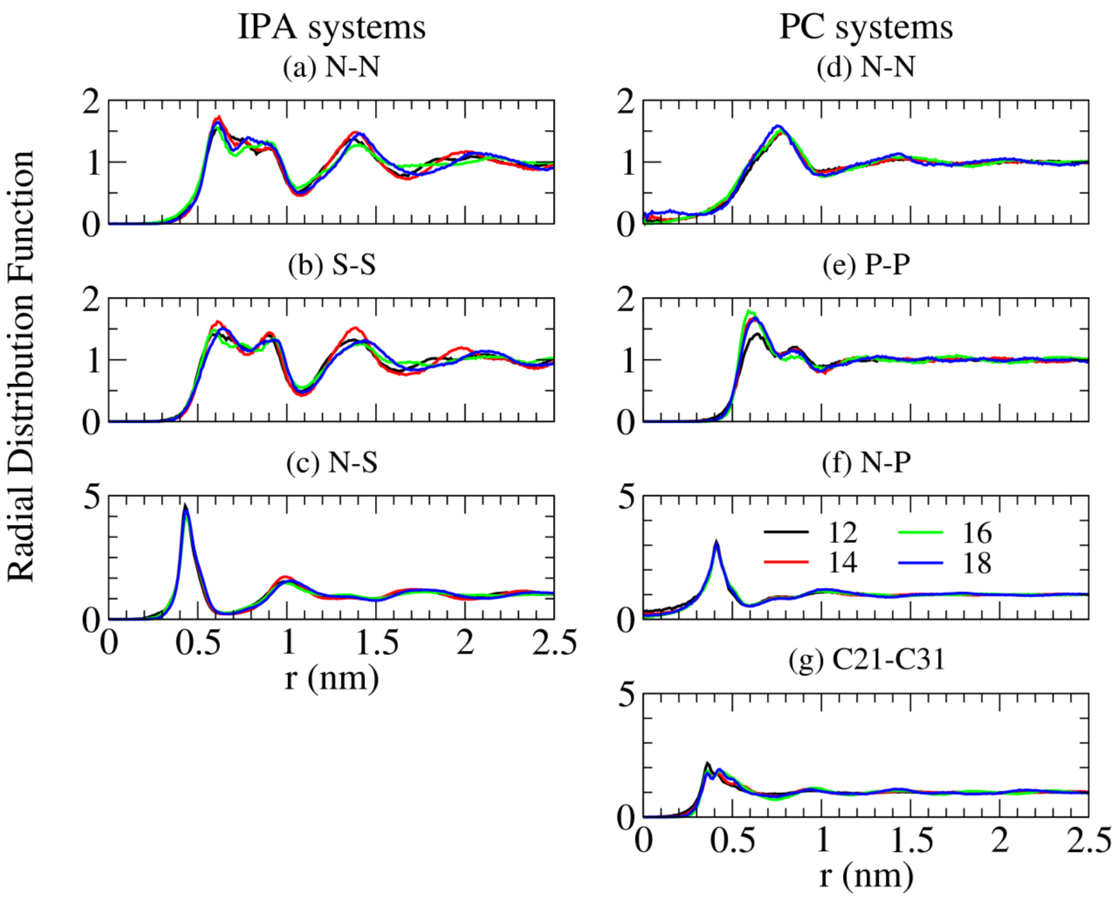

Figure S2 Two-dimensional radial distribution function of the (a) N-N pairs, (b) S-S pairs, and (c) N-S pairs for IPA bilayers and (d) N-N pairs, (e) P-P pairs, (f) N-P pairs, and (g) C21-C31 pairs for PC bilayers at 288 K.

P-N Vector Distributions

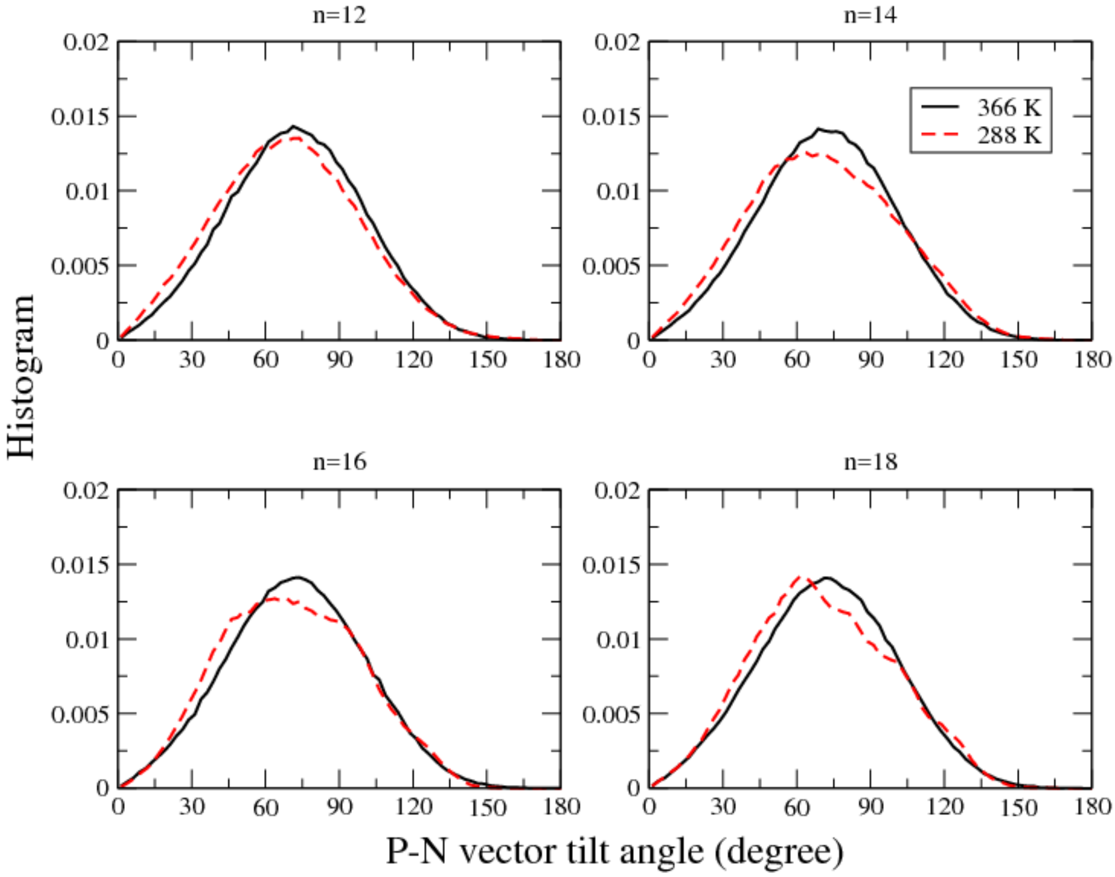

Figure S3 P-N vector distribution for PC bilayers at 366 K (black solid line) and 288 K (red dashed line).

Equlibrium Check

The 100 ns MD equilibration run for each biayer system was splited into two 50 ns trajectories. The bilayer properties of membrane area distributions (Fig. S1) and alkyl chain tilt angle distributions (Fig. S2) were compared to validate the equilibration of the system. The consistent results from two 50 ns trajectories indeed suggested that all systems were equilibrated for the further analyses illustrated in the main text.

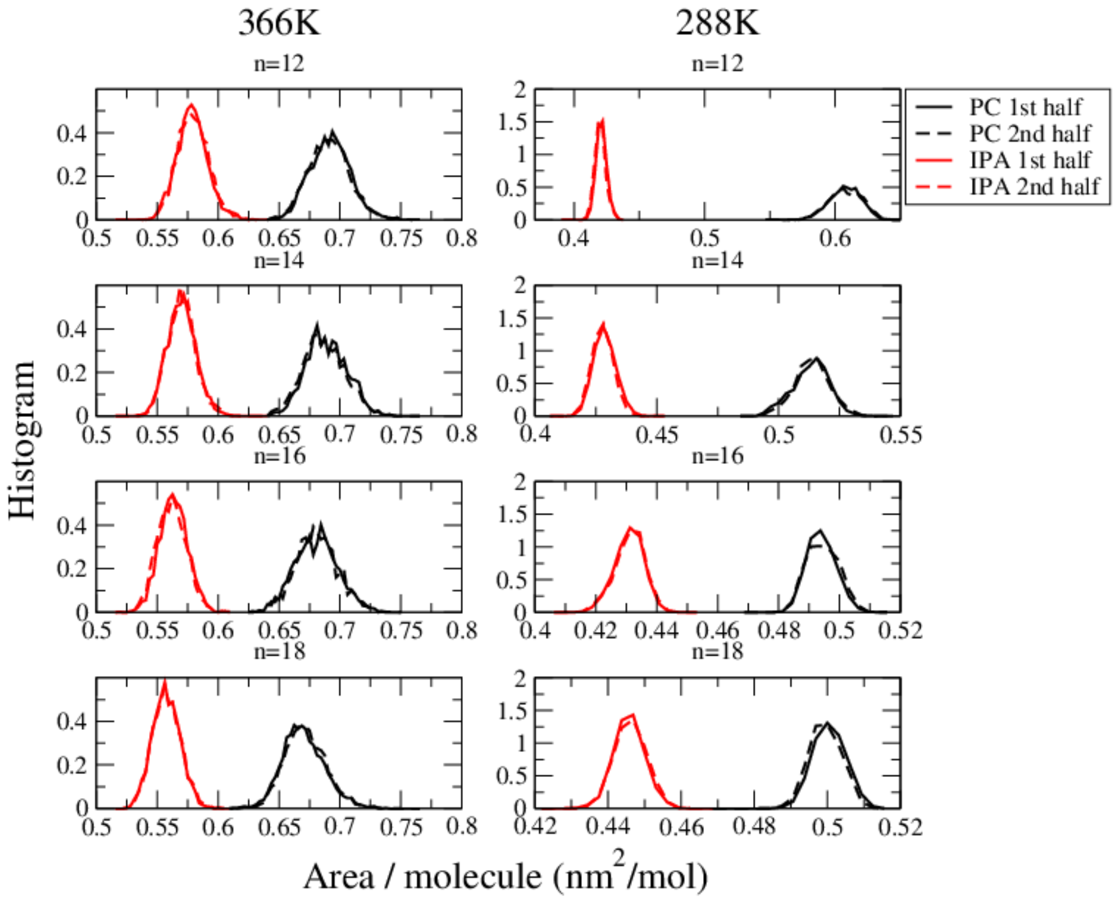

Figure S4 The area distributions for each IPA(red) or PC(black) bilayer system from the first 50 ns (solid line) and the second 50 ns MD runs (dashed line).

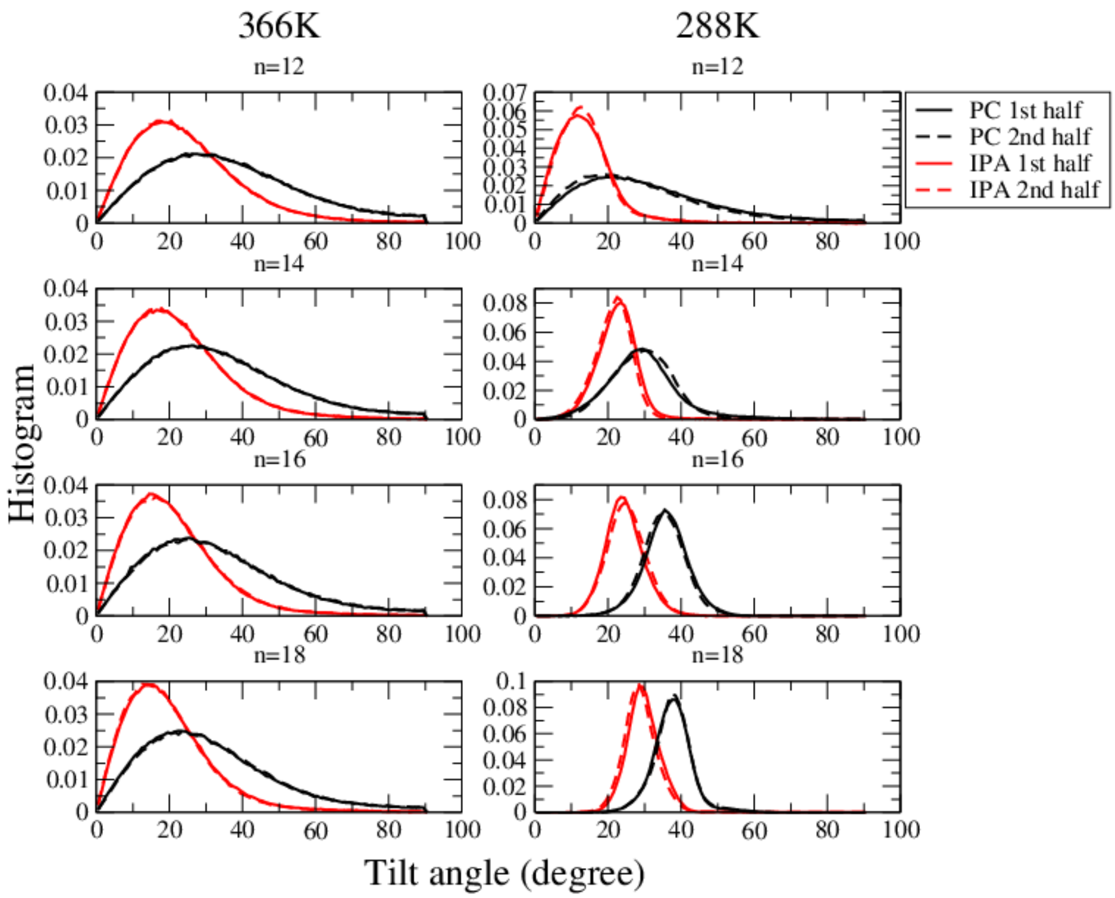

Figure S5 The alkyl chain tilt angle distributions for each IPA(red) or PC(black) bilayer system from the first 50 ns (solid line) and the second 50 ns MD runs (dashed line).

Supporting reference

1. Y. Sugita, Y. Okamoto, 1999. Replica-exchange molecular dynamics method for protein folding, *Chem. Phys. Letters* 314:141–151.

2. D. J. Earla, M. W. Deema, 2005. Parallel Tempering: Theory, Applications, and New Perspectives, *Phys. Chem. Chem. Phys*. 7:3910-3916.
